# Primary sternum diffuse large B-cell lymphoma: A case report and a review of the literatur

**DOI:** 10.3892/ol.2021.12453

**Published:** 2021-01-07

**Authors:** Meng-Ying Tong, Xian Zhang, Zhe Yu, Xiu-Hua Sun, Shuang Li, Yang Zhang

Oncol Lett 9: 2623-2628, 2015; DOI: 10.3892/ol.2015.3122

Subsequently to the publication of the above paper, an interested reader drew to the authors’ attention that the data shown in Fig. 4I and J on p. 2625 for the CD5 and CD3 experiments appeared to be overlapping if one of the panels were rotated through 180°.

The authors have re-examined their data, and realized that the same data panel was inadvertently incorporated into Fig. 4 twice, albeit in the different positions shown. To rectify this situation, and to better illustrate their findings, the authors have requested that they show all their markers in a revised version of Fig. 4, and this is presented on the next page. Furthermore, the legend for Fig. 4 has been revised to the following: “Figure 4. Diffuse infiltration of large B-cells. (A and B) Hematoxylin and eosin-stained cells (A, ×200; B, ×400). Immunohistochemical analysis revealed that the neoplastic cells were positive for (C) CD20, (D) CD79a, (E) BCL-6, (F) CD10, (G) MUM-1 and (H) Ki-67, partially positive for (I) EMA, weakly positive for (J) CD5, (K) CD3 and (L) BCL-2, and negative for (M) AE1/AE3, (N) CD30 and (O) ALK (×400). MUM-1, multiple myeloma oncogene 1; EMA, epithelial membrane antigen; BCL, B-cell lymphoma; AE, anion exchanger; ALK, anaplastic lymphoma kinase.”. Finally, the text in the Results section on p. 2624, line 2, should have read as follows (changes highlighted in bold): “However, the specimen was **weakly positive** for CD5, CD3 a**nd BCL-2**, and **negative for anaplastic lymphoma kinase, CD30 and anion exchanger (AE)1/AE3.**”.

The authors regret the error that was made in the preparation of the published figure, and confirm that this error, together with the revisions now made to the text, did not affect the diagnosis of this case. The authors are grateful to the editor of *Oncology Letters* for allowing them the opportunity to publish a Corrigendum, and all the authors agree to this Corrigendum. Furthermore, they apologize to the readership for any inconvenience caused.

## Figures and Tables

**Figure 4.**
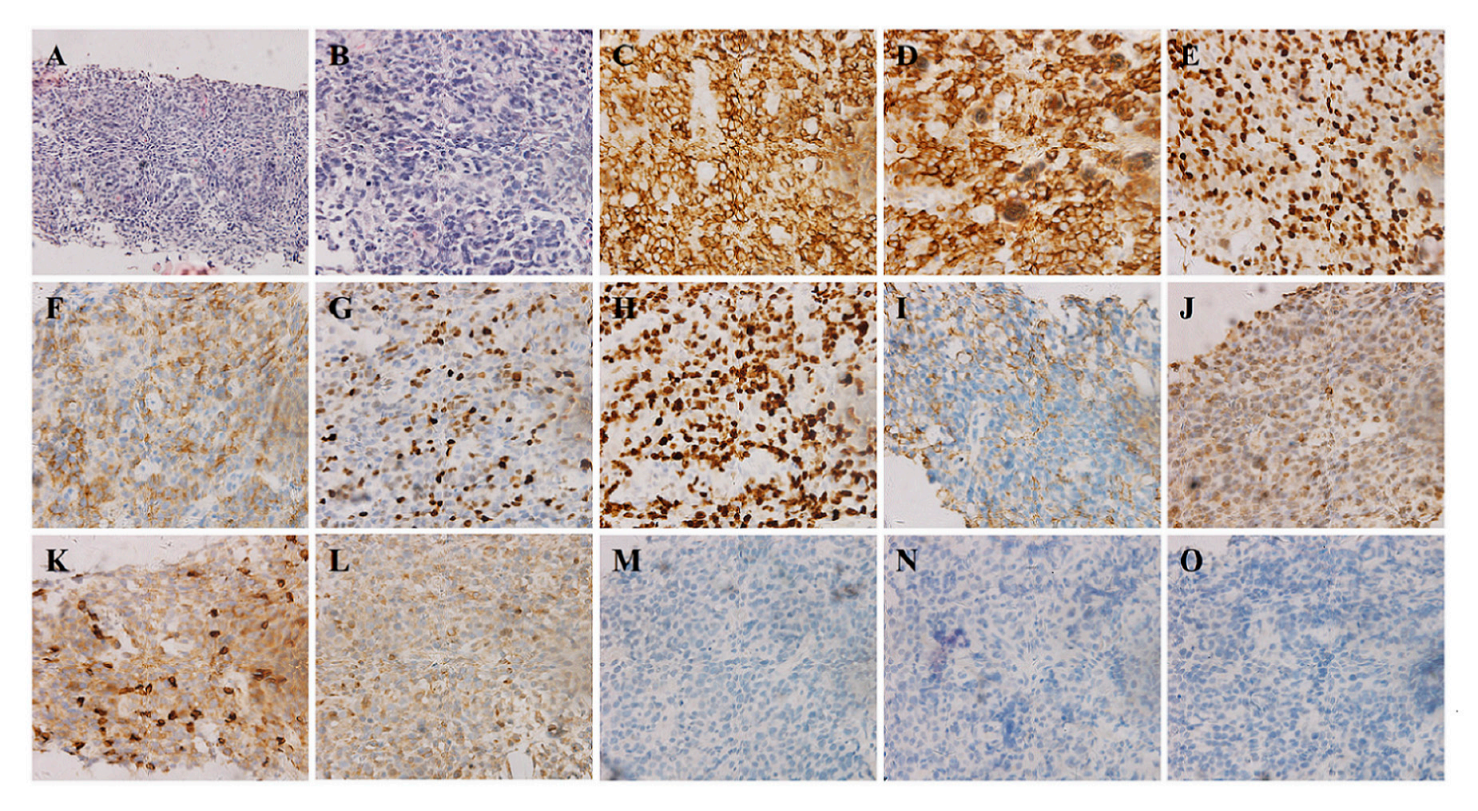
Diffuse infiltration of large B-cells. (A and B) Hematoxylin and eosin-stained cells (A, ×200; B, ×400). Immunohistochemical analysis revealed that the neoplastic cells were positive for (C) CD20, (D) CD79a, (E) BCL-6, (F) CD10, (G) MUM-1 and (H) Ki-67, partially positive for (I) EMA, weakly positive for (J) CD5, (K) CD3 and (L) BCL-2, and negative for (M) AE1/AE3, (N) CD30 and (O) ALK (×400). MUM-1, multiple myeloma oncogene 1; EMA, epithelial membrane antigen; BCL, B-cell lymphoma; AE, anion exchanger; ALK, anaplastic lymphoma kinase.

